# Comparison of the miRNA expression profiles in fresh frozen and formalin-fixed paraffin-embedded tonsillar tumors

**DOI:** 10.1371/journal.pone.0179645

**Published:** 2017-06-23

**Authors:** Zuzana Vojtechova, Jiri Zavadil, Jan Klozar, Marek Grega, Ruth Tachezy

**Affiliations:** 1 Department of Genetics and Microbiology, Faculty of Science, Charles University, Prague, Czech Republic; 2 Department of Immunology, Institute of Hematology and Blood Transfusion, Prague, Czech Republic; 3 Molecular Mechanisms and Biomarkers Group, International Agency for Research on Cancer, Lyon, France; 4 Department of Otorhinolaryngology and Head and Neck Surgery, 1^st^ Faculty of Medicine, Charles University, Motol University Hospital, Prague, Czech Republic; 5 Department of Pathology and Molecular Medicine, 2^nd^ Faculty of Medicine, Charles University, Prague, Czech Republic; University of Massachusetts Medical School, UNITED STATES

## Abstract

MicroRNAs are considered as promising prognostic and diagnostic biomarkers of human cancer since their profiles differ between tumor types. Most of the tumor profiling studies were performed on rarely available fresh frozen (FF) samples. Alternatively, archived formalin-fixed paraffin-embedded (FFPE) tissue samples are also well applicable to larger-scale retrospective miRNA profiling studies. The aim of this study was to perform systematic comparison of the miRNA expression profiles between FF and macrodissected FFPE tonsillar tumors using the TaqMan Low Density Array system, with the data processed by different software programs and two types of normalization methods. We observed a marked correlation between the miRNA expression profiles of paired FF and FFPE samples; however, only 27-38% of the differentially deregulated miRNAs overlapped between the two source systems. The comparison of the results with regard to the distinct modes of data normalization revealed an overlap in 58–67% of differentially expressed miRNAs, with no influence of the choice of software platform. Our study highlights the fact that for an accurate comparison of the miRNA expression profiles from published studies, it is important to use the same type of clinical material and to test and select the best-performing normalization method for data analysis.

## Introduction

MicroRNAs (miRNAs) are small non coding RNAs (~21 nucleotides) which play an important role in post-trancriptional regulation of gene expression. Their binding with perfect or imperfect complementarity to the 3´ untranslated region of the target mRNA leads either to mRNA cleavage and degradation or to inhibition of mRNA translation [1]. Of interest, Ørom et al. also reported an association of miRNA with the 5´ untranslated region which resulted in the activation of gene expression [2, 3]. MiRNAs are involved in many biological processes such as cellular development, proliferation, differentiation, survival or regulation of apoptosis. The expression of miRNAs is deregulated in human cancer. It has been reported that they can act as tumor suppressors or oncogenes based on their targets. The miRNA expression profiles are specific for malignant and non-malignant tissues, and it has been shown the miRNA profiles differ across tumor types [4]. Many studies focused on the miRNA profiling of tumor types of different origin have been published since miRNAs are considered as promising prognostic and diagnostic biomarkers of human cancer [5–7].

Most of the miRNA profiling studies of tumors were at first performed on the fresh frozen samples (FF) where the RNA is well preserved. However, this type of clinical material is rarely available. The disadvantage of formalin fixation is that it preserves the tissue by creating cross-links between all tissue and molecular components, and these modifications can cause the fragmentation of RNA [8]. However, contrary to mRNA, the stability of miRNAs is not influenced by formalin fixation, probably due to their small size and secondary structure [8–10]. Archived material, formalin-fixed paraffin-embedded tissue samples (FFPE), has proven usable for miRNA analyses and allows for retrospective large studies where the results can be correlated with clinical parameters and prognosis of patients.

Numerous studies have shown the tissue specific expression profiles as well as the differences in the abundantly expressed miRNAs in different types of tumors. However, the overlap of the lists of tumor specific miRNAs is, for most cancer types, poor. Several reasons can explain the discrepant data. One of them can be the use of different types of clinical material or of different methodological approaches.

In our previously published study [11], we found that the tumor homogeneity is important for the robustness of miRNA expression studies, especially in head and neck tumors which are very heterogeneous. Therefore, macrodissection of the FFPE samples provides benefit by increasing the homogeneity of the analyzed samples. To evaluate our previous data on FF HNC samples, we analyzed paired FF and macrodissected FFPE samples for the abundantly expressed miRNAs and the level of concordance in the profiles.

Few studies comparing miRNA expression in FF and FFPE paired samples have been published [12–14]. In the majority of microarray studies, good correlation of results was revealed (correlation coefficient up to 0.95), but the studies were either based on small groups of samples or differed in the workflow. None of these comparative studies has been conducted in head and neck tumors.

The aims of our study were to perform the miRNA expression profiling in a set of macrodissected FFPE samples, to compare the results with the miRNA profiles of paired fresh frozen tumors, and to assess differences possibly resulting from the use of different normalization methods and software for data analysis. Finally, from the analyses of FFPE samples, several differentially expressed miRNAs were selected for confirmation in a larger set of macrodissected FFPE tissues.

## Materials and methods

### Clinical samples

Ten cases of tonsillar tumors and five non-malignant tonsillar tissues were selected for the comparison of the miRNA expression profiles in fresh frozen and formalin-fixed paraffin embedded samples. All samples were obtained from patients treated at the Department of Otolaryngology and Head and Neck Surgery, 1^st^ Faculty of Medicine, Charles University and Motol University Hospital in Prague. Fresh-frozen samples were already used in our recent study [11]. The signed informed consent form was obtained from all patients enrolled in this study. The study received official institutional and ethical approval from the Motol University Hospital and Institute of Hematology and Blood Transfusion. The study set was selected based on the presence of HPV DNA and HPV E6 mRNA we defined in our previous studies [11, 15].

The sampling and tissue handling for FF samples has been described before [11, 15]. Sections of FF tumor samples were cut on a cryostat, and the number of tumor cells was determined by a pathologist. All FF samples contained more than 50% of tumor cells. FFPE samples were macrodissected as described before [11].

### Processing of samples

Total RNA from all FF tissues was isolated by the miRVana kit (Life Technologies, USA) according to the manufacturer's protocol. Total RNA from FFPE samples was isolated simultaneously with DNA from four 10-μm sections enriched for the tumor cells by macrodissection using the Ambion RecoverAll^™^ Total Nucleic Acid Isolation kit for FFPE according to the manufacturer's protocol (Applied Biosystems, USA). RNA concentration and quality was measured by a Nanodrop^™^ Spectrophotometer (Thermo Scientific, USA) and Experion chip electrophoresis (Bio-Rad, USA).

### TaqMan Low Density Array (TLDA) analysis

The miRNA expression profiling was performed by the TaqMan^®^ Array Human MicroRNA A+B Cards Set v3.0 (Life Technologies, USA) containing a total of 384 miRNA probes and controls per card. Overall, we analyzed 15 FF samples and 15 FFPE samples (five HPV-positive tumor samples, five HPV-negative tumor samples, and five non-malignant tissues of each material). For the analysis of FF samples, 1000 ng of total RNA was used for reverse transcription. In the case of FFPE samples, the input was 350 ng due to the smaller concentration of extracted RNA from FFPE samples. Total RNA of each sample was reverse transcribed using the TaqMan^®^ MicroRNA Reverse Transcription Kit and Megaplex^™^ RT Primers specific for each card (both Life Technologies, USA) according to the manufacturer's protocol. The workflow without preamplification was chosen for both types of material, FF as well as FFPE samples. The TLDA cards were analyzed on the Applied Biosystems 7900HT Real-Time PCR System.

### Data analysis

Each set of samples was evaluated separately, and the results were compared. For data processing and evaluation, we used the SDS 2.4 and the ExpressionSuite v1.0.1 software (Life Technologies, USA). Ct values identified by automated thresholding were exported separately for cards A and B, and the RQ (relative quantity) was calculated from the detected Ct values using the 2^-Ct^ formula. Data were further processed by the GeneSpring GX v13.1 software (Agilent), GenEx v6.1 (MultiD), and MeV v4.9 (TIGR TM4 suite) for comparison. We also applied two types of data normalization—50^th^ percentile shift and global normalization—to perform within-sample normalization, globally across the data sets. For comparison of two groups in censored analyses, the T-test in combination with Pavlidis Template Matching (PTM) algorithm were used. In the comparison of differential abundance of miRNAs in the groups, the *P*-value (*P*<0.05) and fold-change (FC>1.33) were set. Only the miRNAs differentially expressed in at least 3/5 or 6/10 (60%) samples with measured results were considered for further analyses. The correlation was evaluated in the GraphPad InStat 3.0 tool using the Spearman nonparametric correlation.

### Confirmation of microarray results

Selected miRNAs which were found to be differentially expressed by the microarrays in FFPE tonsillar tumor samples in comparison to the non-malignant tonsillar tissues were confirmed by the individual RT qPCR using the TaqMan^®^ MicroRNA Assays (Life Technologies, USA) as described recently [11]. Total RNA from five FFPE samples of non-malignant tonsils were used as a calibrator, and RNU48 served as the endogenous control. The data were analyzed in the GenEx v6.1 (MultiD) software. The 2^-ΔΔCt^ method was used for calculations of the fold change. The cut-off fold change was set as for the arrays to +/- 1.33 (33% fold change). T-test or Mann Whitney nonparametric test was applied depending on the data distribution. All results were statistically significant (P-value ≤0.05).

## Results

**Fig 1:**
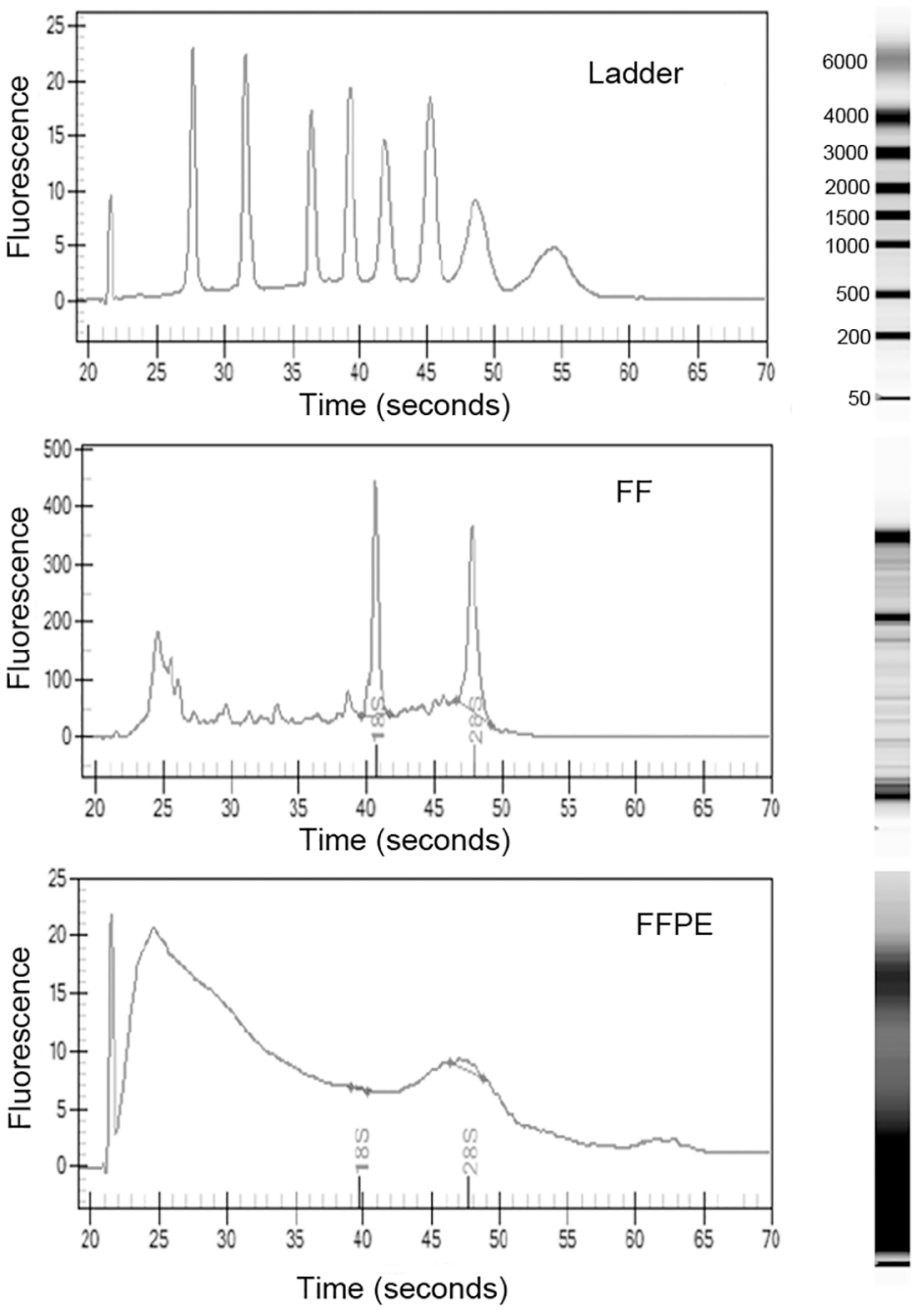
Comparison of electropherogram outputs. At the top—ladder, in the middle—fresh frozen sample, at the bottom—formalin-fixed paraffin-embedded sample. Right—lines from virtual gel.

The quality and concentration of total RNA was analyzed by a Nanodrop^™^ Spectrophotometer and Experion chip electrophoresis. The RNA integrity number (RIN) was higher than seven in all FF samples. As documented in Fig 1, total RNA isolated from FFPE samples showed characteristic profiles for degraded RNA fragments (RIN in the range 1.9–2.6), but fragments with a length of around 200 bp were present allowing for a miRNA expression analyses.

**Fig 2:**
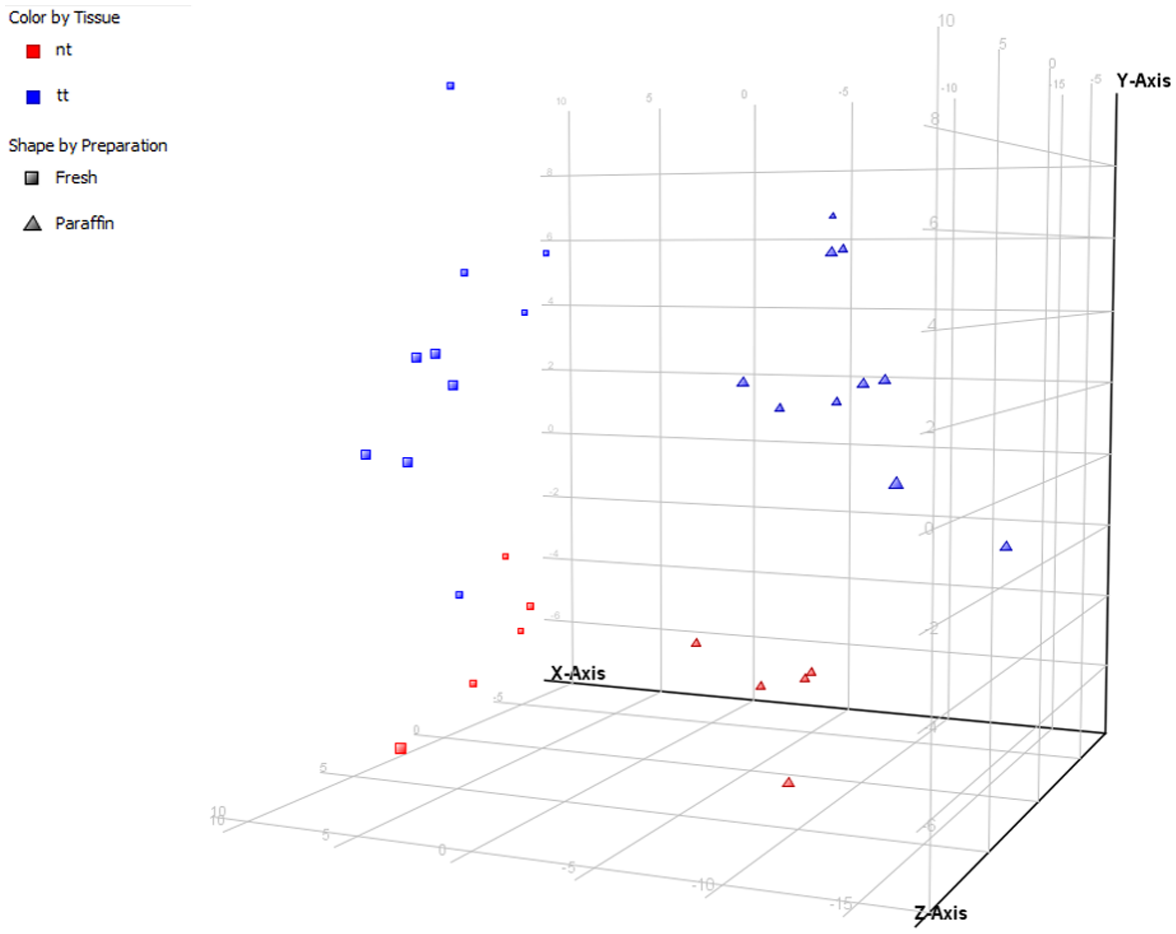
Principal component analysis plot. Visualization of the miRNA expression in fresh frozen (squares) and formalin-fixed paraffin-embedded (triangles) samples, as well as miRNA expression separation of tumor (blue) and non-malignant samples (red).

**Fig 3:**
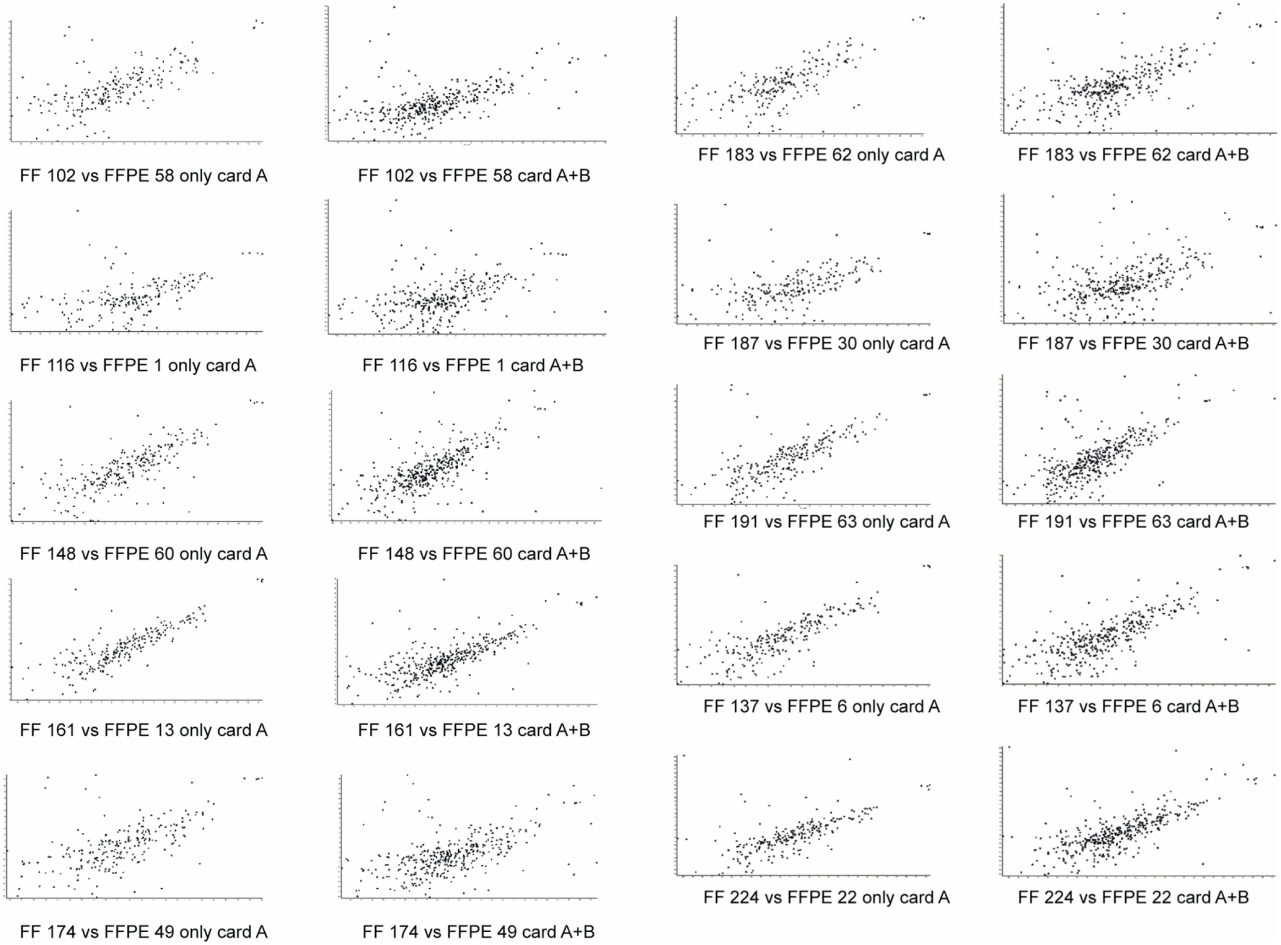
Correlation of paired samples visualized on scatterplots. Scatter plots of the normalized RQ values in pair-wise comparison from fresh-frozen (FF) and formalin-fixed paraffin embedded (FFPE) samples.

As illustrated by the principal component analysis (PCA) (Fig 2), the miRNA expression profiles of all samples differ depending on the analyzed type of material (FF vs FFPE) and characteristics of the tissue, tumor vs normal tissue (TT vs NT). The correlation between the miRNA expression profiles of paired FF and FFPE samples was evaluated using the Spearman correlation test. The correlation of the expression level for the well characterized miRNAs located on card A was better (correlation coefficient ≥0.65 in 7/10 samples (p<0.0001)) in comparison to all analyzed miRNAs (miRNAs on cards A and B). The exact Spearman correlation values of each comparison and the confidence intervals are summarized in Table 1. The correlation of paired samples is also visualized in scatter plots (Fig 3). Our assumption that FF samples with higher percentage of tumor cells would be more likely to give better correlation of the expression miRNA profiles with FFPE macrodissected samples was not proven. The correlation coefficient of the miRNA expression profiles of FF and FFPE samples was not influenced by the number of tumor cells in the particular FF sample (Table 1).

**Table 1:** Spearman correlation values between miRNA expression profiles of FF and FFPE paired samples.

Sample		R	CI	tumor cells in FF (%)
**FF 102 vs FFPE 58**	card A	0,6982	0,6199 to 0,7628	> 50%
	card A+B	0,5848	0,5088 to 0,6517	
**FF 116 vs FFPE 1**	card A	0,5194	0,4064 to 0,6167	> 70%
	card A+B	0,4642	0,3698 to 0,5491	
**FF 148 vs FFPE 60**	card A	0,7406	0,6757 to 0,7941	> 70%
	card A+B	0,6749	0,6150 to 0,7271	
**FF 161 vs FFPE 13**	card A	0,8302	0,7829 to 0,8680	> 60%
	card A+B	0,7174	0,6632 to 0,7641	
**FF 174 vs FFPE 49**	card A	0,6378	0,5500 to 0,7117	> 50%
	card A+B	0,5748	0,4999 to 0,6412	
**FF 183 vs FFPE 62**	card A	0,6905	0,6098 to 0,7570	70%
	card A+B	0,6373	0,5664 to 0,6989	
**FF 187 vs FFPE 30**	card A	0,5879	0,4883 to 0,6724	> 70%
	card A+B	0,5152	0,4289 to 0,5922	
**FF 191 vs FFPE 63**	card A	0,7332	0,6648 to 0,7894	> 80%
	card A+B	0,6593	0,5959 to 0,7145	
**FF 137 vs FFPE 6**	card A	0,7914	0,7363 to 0,8360	90%
	card A+B	0,7361	0,6828 to 0,7816	
**FF 224 vs FFPE 22**	card A	0,7371	0,6697 to 0,7925	80%
	card A+B	0,7124	0,6571 to 0,7601	

Differentially expressed miRNAs were identified by the *P*-value (*P*<0.05) and fold-change >1.33 with the 50^th^ percentile shift normalization method using the GeneSpring software. In our study, macrodissected FFPE samples revealed less differentially expressed miRNAs (N = 58) than paired fresh frozen tumors (N = 83 miRNAs). When comparing the lists of deregulated miRNAs between FF and FFPE samples, we detected overlaps in 27-38% (Table 2, on the left).

**Table 2:** Differentially expressed miRNAs detected in tonsillar tumors versus non-malignant tonsillar tissue. The overlap between FF vs FFPE material is highlighted in green. On the left—results using 50th percentile shift normalization for data analysis. On the right—results using global normalization for data analysis. FC—fold change.

50th percentile shift normalization				global normalization			
FF		FFPE		FF		FFPE	
TTall vs NT				TTall vs NT			
miRNA	FC	miRNA	FC	miRNA	FC	miRNA	FC
hsa-miR-101-002253	-2.87	hsa-miR-101-002253	-8.70	hsa-let-7g-002282	-1.85	hsa-let-7g-002282	-2.57
hsa-miR-126#-000451	-2.29	hsa-miR-126#-000451	-8.27	hsa-miR-126#-000451	-1.85	hsa-miR-126#-000451	-4.28
hsa-miR-130b-000456	1.86	hsa-miR-130b-000456	3.42	hsa-miR-140-3p-002234	-2.10	hsa-miR-140-3p-002234	-4.50
hsa-miR-140-3p-002234	-2.42	hsa-miR-140-3p-002234	-4.15	hsa-miR-141-000463	4.71	hsa-miR-141-000463	3.85
hsa-miR-141-000463	4.04	hsa-miR-141-000463	2.67	hsa-miR-143-002249	-2.14	hsa-miR-143-002249	-1.68
hsa-miR-142-3p-000464	-3.85	hsa-miR-142-3p-000464	-8.79	hsa-miR-144#-002148	-4.52	hsa-miR-144#-002148	-2.95
hsa-miR-143-002249	-2.02	hsa-miR-143-002249	-2.89	hsa-miR-184-000485	-6.15	hsa-miR-184-000485	-5.46
hsa-miR-195-000494	-1.92	hsa-miR-195-000494	-2.89	hsa-miR-196b-002215	7.00	hsa-miR-196b-002215	2.88
hsa-miR-196b-002215	8.26	hsa-miR-196b-002215	4.42	hsa-miR-200a-000502	3.52	hsa-miR-200a-000502	3.32
hsa-miR-200b-002251	3.86	hsa-miR-200b-002251	2.60	hsa-miR-200b-002251	3.04	hsa-miR-200b-002251	2.89
hsa-miR-200c-002300	3.45	hsa-miR-200c-002300	3.00	hsa-miR-200c-002300	2.83	hsa-miR-200c-002300	3.33
hsa-miR-205-000509	4.22	hsa-miR-205-000509	5.12	hsa-miR-205-000509	4.58	hsa-miR-205-000509	6.71
hsa-miR-21#-002438	2.99	hsa-miR-21#-002438	2.53	hsa-miR-210-000512	3.74	hsa-miR-210-000512	3.26
hsa-miR-210-000512	4.10	hsa-miR-210-000512	2.70	hsa-miR-224-002099	5.62	hsa-miR-224-002099	5.78
hsa-miR-221-000524	2.69	hsa-miR-221-000524	2.70	hsa-miR-27a#-002445	4.10	hsa-miR-27a#-002445	2.79
hsa-miR-224-002099	6.01	hsa-miR-224-002099	6.03	hsa-miR-27a-000408	2.19	hsa-miR-27a-000408	1.80
hsa-miR-27a#-002445	3.12	hsa-miR-27a#-002445	4.47	hsa-miR-29a-002112	-1.61	hsa-miR-29a-002112	-2.57
hsa-miR-27a-000408	1.81	hsa-miR-27a-000408	1.92	hsa-miR-429-001024	3.25	hsa-miR-429-001024	2.37
hsa-miR-29a-002112	-1.83	hsa-miR-29a-002112	-3.26	hsa-miR-452-002329	2.79	hsa-miR-452-002329	1.89
hsa-miR-452-002329	3.00	hsa-miR-452-002329	3.84	hsa-miR-484-001821	2.02	hsa-miR-484-001821	2.40
hsa-miR-486-3p-002093	-3.79	hsa-miR-486-3p-002093	-4.97	hsa-miR-886-3p-002194	3.04	hsa-miR-886-3p-002194	2.94
hsa-miR-886-3p-002194	3.00	hsa-miR-886-3p-002194	2.59	hsa-let-7i#-002172	-2.59	hsa-miR-106b-000442	1.94
hsa-miR-100-000437	-1.62	hsa-let-7b#-002404	-6.10	hsa-miR-100-000437	-1.88	hsa-miR-1253-002894	11.36
hsa-miR-1180-002847	3.95	hsa-let-7g-002282	-3.04	hsa-miR-101-002253	-2.43	hsa-miR-1260-002896	1.58
hsa-miR-1227-002769	4.37	hsa-miR-126-002228	-3.28	hsa-miR-1244-002791	2.49	hsa-miR-126-002228	-2.95
hsa-miR-125b-000449	-1.93	hsa-miR-1262-002852	-10500.89	hsa-miR-125b-000449	-2.44	hsa-miR-127-000452	3.13
hsa-miR-132-000457	1.66	hsa-miR-127-000452	2.01	hsa-miR-132-000457	1.75	hsa-miR-1282-002803	1.35
hsa-miR-135b#-002159	5.18	hsa-miR-138-002284	-3.48	hsa-miR-135b-002261	4.30	hsa-miR-130b-000456	3.51
hsa-miR-135b-002261	4.25	hsa-miR-146a-000468	-3.45	hsa-miR-138-002284	-3.48	hsa-miR-142-3p-000464	-5.87
hsa-miR-136#-002100	-3.36	hsa-miR-146b-001097	-2.48	hsa-miR-139-5p-002289	-3.48	hsa-miR-142-5p-002248	-2.40
hsa-miR-139-3p-002313	-2.80	hsa-miR-150-000473	-10.82	hsa-miR-141#-002145	2.64	hsa-miR-146a-000468	-2.75
hsa-miR-139-5p-002289	-2.97	hsa-miR-155-002623	-3.39	hsa-miR-145#-002149	-2.09	hsa-miR-146b-001097	-2.25
hsa-miR-144#-002148	-5.50	hsa-miR-15a-000389	-2.18	hsa-miR-145-002278	-2.28	hsa-miR-150-000473	-8.31
hsa-miR-145#-002149	-2.55	hsa-miR-16-000391	-2.58	hsa-miR-146b-3p-002361	1.29	hsa-miR-152-000475	2.37
hsa-miR-145-002278	-2.60	hsa-miR-188-3p-002106	-15.67	hsa-miR-151-3p-002254	1.69	hsa-miR-155-002623	-3.37
hsa-miR-181a-000480	-1.50	hsa-miR-19a-000395	-2.14	hsa-miR-151-5P-002642	-1.96	hsa-miR-16-000391	-2.23
hsa-miR-182-002334	3.36	hsa-miR-19b-000396	-2.64	hsa-miR-15b#-002173	4.90	hsa-miR-183#-002270	1.77
hsa-miR-1825-002907	8.50	hsa-miR-20a#-002437	-4.90	hsa-miR-15b-000390	1.89	hsa-miR-18a#-002423	1.84
hsa-miR-183-002269	2.77	hsa-miR-21-000397	1.49	hsa-miR-181a-000480	-1.64	hsa-miR-19b-000396	-2.58
hsa-miR-184-000485	-22.46	hsa-miR-213-000516	-4.21	hsa-miR-183-002269	2.49	hsa-miR-20a#-002437	-2.15
hsa-miR-191#-002678	4.11	hsa-miR-219-2-3p-002390	26.64	hsa-miR-195-000494	-2.33	hsa-miR-21#-002438	2.58
hsa-miR-197-000497	1.72	hsa-miR-323-3p-002227	5.88	hsa-miR-199a-3p-002304	-1.59	hsa-miR-213-000516	-3.40
hsa-miR-200a#-001011	3.42	hsa-miR-342-3p-002260	-3.35	hsa-miR-200a#-001011	4.35	hsa-miR-221-000524	2.86
hsa-miR-200a-000502	3.64	hsa-miR-342-5p-002147	-3.96	hsa-miR-21#-002438	3.76	hsa-miR-223#-002098	-2.63
hsa-miR-204-000508	-5.28	hsa-miR-362-001273	2.43	hsa-miR-21-000397	2.68	hsa-miR-299-5p-000600	4.75
hsa-miR-211-000514	34.58	hsa-miR-374-000563	-2.24	hsa-miR-214#-002293	-1.74	hsa-miR-320-002277	1.92
hsa-miR-222#-002097	2.09	hsa-miR-423-5p-002340	-5.05	hsa-miR-222#-002097	2.38	hsa-miR-323-3p-002227	4.17
hsa-miR-24-000402	1.59	hsa-miR-455-3p-002244	7.08	hsa-miR-222-002276	2.04	hsa-miR-342-3p-002260	-3.14
hsa-miR-26a-000405	-2.12	hsa-miR-485-3p-001277	3.99	hsa-miR-24-000402	1.73	hsa-miR-362-001273	2.07
hsa-miR-26b#-002444	-5.11	hsa-miR-490-001037	3.37	hsa-miR-26a-000405	-1.84	hsa-miR-455-3p-002244	8.08
hsa-miR-26b-000407	-2.22	hsa-miR-516-3p-001149	-12.45	hsa-miR-26b#-002444	-3.16	hsa-miR-485-3p-001277	2.33
hsa-miR-27b#-002174	1.88	hsa-miR-566-001533	-4.10	hsa-miR-26b-000407	-2.85	hsa-miR-532-001518	-2.24
hsa-miR-29a#-002447	-2.62	hsa-miR-583-001623	12.93	hsa-miR-29a#-002447	-1.98	hsa-miR-550-002410	-2.05
hsa-miR-29b-000413	-2.17	hsa-miR-590-5p-001984	-6.36	hsa-miR-29b-000413	-2.45	hsa-miR-590-5p-001984	-2.27
hsa-miR-29c-000587	-2.39	hsa-miR-640-001584	-4.53	hsa-miR-29c-000587	-2.64	hsa-miR-629-001562	-2.19
hsa-miR-302a-000529	222.12	hsa-miR-660-001515	-1.96	hsa-miR-302c-000533	18.15	hsa-miR-663B-002857	1.78
hsa-miR-30a-3p-000416	-3.27	hsa-miR-663B-002857	2.55	hsa-miR-30b-000602	-1.76	hsa-miR-888-002212	-551.31
hsa-miR-30a-5p-000417	-1.93	hsa-miR-942-002187	-3.76	hsa-miR-30d-000420	-1.95		
hsa-miR-30d-000420	-1.94			hsa-miR-31-002279	6.09		
hsa-miR-30e-3p-000422	-2.39			hsa-miR-32-002109	-2.47		
hsa-miR-31-002279	5.72			hsa-miR-335#-002185	2.58		
hsa-miR-335#-002185	2.04			hsa-miR-342-5p-002147	-7.89		
hsa-miR-335-000546	2.33			hsa-miR-34b-002102	2.35		
hsa-miR-34a#-002316	1.91			hsa-miR-505#-002087	-1.68		
hsa-miR-378-000567	-1.87			hsa-miR-511-001111	2.16		
hsa-miR-429-001024	3.66			hsa-miR-520g-001121	3.52		
hsa-miR-431-001979	16.66			hsa-miR-577-002675	-3.38		
hsa-miR-484-001821	2.01			hsa-miR-596-001550	-2.00		
hsa-miR-486-001278	-3.26			hsa-miR-649-001602	3.05		
hsa-miR-497-001043	-4.12			hsa-miR-708-002341	2.14		
hsa-miR-505#-002087	-2.84			hsa-miR-944-002189	5.27		
hsa-miR-511-001111	3.25			hsa-miR-9-000583	7.17		
hsa-miR-517a-002402	3.23						
hsa-miR-517c-001153	3.66						
hsa-miR-523-002386	10.93						
hsa-miR-576-3p-002351	8.52						
hsa-miR-627-001560	29.22						
hsa-miR-643-001594	2.71						
hsa-miR-658-001513	2.12						
hsa-miR-659-001514	6.10						
hsa-miR-708-002341	2.26						
hsa-miR-944-002189	6.72						
hsa-miR-99b#-002196	-4.26						
**22/83 (27%)**		**22/58 (38%)**		**21/72 (29%)**		**21/57 (37%)**	

**Fig 4:**
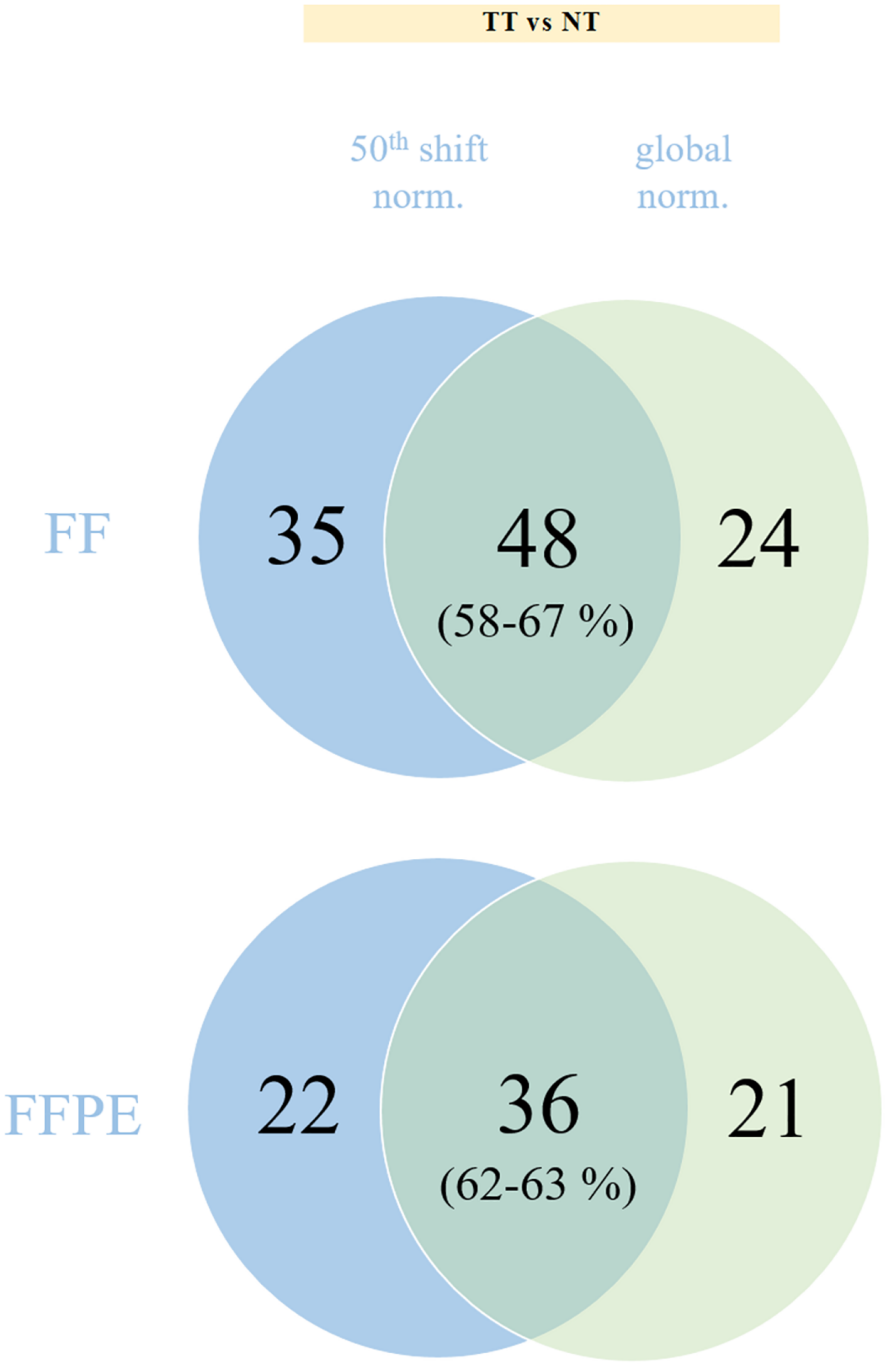
Comparison of differentially expressed miRNAs. Comparison of differentially expressed miRNAs identified using 50^th^ percentile shift normalization and global normalization for each clinical material.

To establish how the method used for normalization influences the final results, we applied, besides the 50^th^ percentile shift normalization, also global normalization for each group of the analyzed samples. As illustrated in Fig 4 and Table 3, the differentially expressed miRNAs based on each normalization method overlap in 58–67% for FF and/or FFPE samples. Using different software and the identical normalization method, almost 90% of the deregulated miRNAs were concordant between FF and FFPE samples (data not shown).

**Table 3:** Differentially expressed miRNAs detected tonsillar tumors versus non-malignant tonsillar tissue. Overlap between results using 50th percentile shift normalization and global normalization is highlighted in green. On the left—results from FF samples, on the right—results from FFPE samples. FC—fold change.

FF				FFPE			
TTall vs NT				TTall vs NT			
50th percentile shift normalization		global normalization		50th percentile shift normalization		global normalization	
miRNA	FC	miRNA	FC	miRNA	FC	miRNA	FC
hsa-miR-100-000437	-1.62	hsa-miR-100-000437	-1.88	hsa-let-7g-002282	-3.04	hsa-let-7g-002282	-2.57
hsa-miR-101-002253	-2.87	hsa-miR-101-002253	-2.43	hsa-miR-126#-000451	-8.27	hsa-miR-126#-000451	-4.28
hsa-miR-125b-000449	-1.93	hsa-miR-125b-000449	-2.44	hsa-miR-126-002228	-3.28	hsa-miR-126-002228	-2.95
hsa-miR-126#-000451	-2.29	hsa-miR-126#-000451	-1.85	hsa-miR-127-000452	2.01	hsa-miR-127-000452	3.13
hsa-miR-132-000457	1.66	hsa-miR-132-000457	1.75	hsa-miR-130b-000456	3.42	hsa-miR-130b-000456	3.51
hsa-miR-135b-002261	4.25	hsa-miR-135b-002261	4.30	hsa-miR-140-3p-002234	-4.15	hsa-miR-140-3p-002234	-4.50
hsa-miR-139-5p-002289	-2.97	hsa-miR-139-5p-002289	-3.48	hsa-miR-141-000463	2.67	hsa-miR-141-000463	3.85
hsa-miR-140-3p-002234	-2.42	hsa-miR-140-3p-002234	-2.10	hsa-miR-142-3p-000464	-8.79	hsa-miR-142-3p-000464	-5.87
hsa-miR-141-000463	4.04	hsa-miR-141-000463	4.71	hsa-miR-143-002249	-2.89	hsa-miR-143-002249	-1.68
hsa-miR-143-002249	-2.02	hsa-miR-143-002249	-2.14	hsa-miR-146a-000468	-3.45	hsa-miR-146a-000468	-2.75
hsa-miR-144#-002148	-5.50	hsa-miR-144#-002148	-4.52	hsa-miR-146b-001097	-2.48	hsa-miR-146b-001097	-2.25
hsa-miR-145#-002149	-2.55	hsa-miR-145#-002149	-2.09	hsa-miR-150-000473	-10.82	hsa-miR-150-000473	-8.31
hsa-miR-145-002278	-2.60	hsa-miR-145-002278	-2.28	hsa-miR-155-002623	-3.39	hsa-miR-155-002623	-3.37
hsa-miR-181a-000480	-1.50	hsa-miR-181a-000480	-1.64	hsa-miR-16-000391	-2.58	hsa-miR-16-000391	-2.23
hsa-miR-183-002269	2.77	hsa-miR-183-002269	2.49	hsa-miR-196b-002215	4.42	hsa-miR-196b-002215	2.88
hsa-miR-184-000485	-22.46	hsa-miR-184-000485	-6.15	hsa-miR-19b-000396	-2.64	hsa-miR-19b-000396	-2.58
hsa-miR-195-000494	-1.92	hsa-miR-195-000494	-2.33	hsa-miR-200b-002251	2.60	hsa-miR-200b-002251	2.89
hsa-miR-196b-002215	8.26	hsa-miR-196b-002215	7.00	hsa-miR-200c-002300	3.00	hsa-miR-200c-002300	3.33
hsa-miR-200a#-001011	3.42	hsa-miR-200a#-001011	4.35	hsa-miR-205-000509	5.12	hsa-miR-205-000509	6.71
hsa-miR-200a-000502	3.64	hsa-miR-200a-000502	3.52	hsa-miR-20a#-002437	-4.90	hsa-miR-20a#-002437	-2.15
hsa-miR-200b-002251	3.86	hsa-miR-200b-002251	3.04	hsa-miR-21#-002438	2.53	hsa-miR-21#-002438	2.58
hsa-miR-200c-002300	3.45	hsa-miR-200c-002300	2.83	hsa-miR-210-000512	2.70	hsa-miR-210-000512	3.26
hsa-miR-205-000509	4.22	hsa-miR-205-000509	4.58	hsa-miR-213-000516	-4.21	hsa-miR-213-000516	-3.40
hsa-miR-21#-002438	2.99	hsa-miR-21#-002438	3.76	hsa-miR-221-000524	2.90	hsa-miR-221-000524	2.86
hsa-miR-210-000512	4.10	hsa-miR-210-000512	3.74	hsa-miR-224-002099	6.03	hsa-miR-224-002099	5.78
hsa-miR-222#-002097	2.09	hsa-miR-222#-002097	2.38	hsa-miR-27a#-002445	4.47	hsa-miR-27a#-002445	2.79
hsa-miR-224-002099	6.01	hsa-miR-224-002099	5.62	hsa-miR-27a-000408	1.92	hsa-miR-27a-000408	1.80
hsa-miR-24-000402	1.59	hsa-miR-24-000402	1.73	hsa-miR-29a-002112	-3.26	hsa-miR-29a-002112	-2.57
hsa-miR-26a-000405	-2.12	hsa-miR-26a-000405	-1.84	hsa-miR-323-3p-002227	5.88	hsa-miR-323-3p-002227	4.17
hsa-miR-26b#-002444	-5.11	hsa-miR-26b#-002444	-3.16	hsa-miR-342-3p-002260	-3.35	hsa-miR-342-3p-002260	-3.14
hsa-miR-26b-000407	-2.22	hsa-miR-26b-000407	-2.85	hsa-miR-362-001273	2.43	hsa-miR-362-001273	2.07
hsa-miR-27a#-002445	3.12	hsa-miR-27a#-002445	4.10	hsa-miR-452-002329	3.84	hsa-miR-452-002329	1.90
hsa-miR-27a-000408	1.81	hsa-miR-27a-000408	2.19	hsa-miR-455-3p-002244	7.08	hsa-miR-455-3p-002244	8.08
hsa-miR-29a#-002447	-2.62	hsa-miR-29a#-002447	-1.98	hsa-miR-485-3p-001277	3.99	hsa-miR-485-3p-001277	2.33
hsa-miR-29a-002112	-1.83	hsa-miR-29a-002112	-1.61	hsa-miR-590-5p-001984	-6.36	hsa-miR-590-5p-001984	-2.27
hsa-miR-29b-000413	-2.17	hsa-miR-29b-000413	-2.45	hsa-miR-886-3p-002194	2.59	hsa-miR-886-3p-002194	2.94
hsa-miR-29c-000587	-2.39	hsa-miR-29c-000587	-2.64	hsa-let-7b#-002404	-6.10	hsa-miR-106b-000442	1.94
hsa-miR-30d-000420	-1.94	hsa-miR-30d-000420	-1.95	hsa-miR-101-002253	-8.70	hsa-miR-1253-002894	11.36
hsa-miR-31-002279	5.72	hsa-miR-31-002279	6.09	hsa-miR-1262-002852	-10500.89	hsa-miR-1260-002896	1.58
hsa-miR-335#-002185	2.04	hsa-miR-335#-002185	2.58	hsa-miR-138-002284	-3.48	hsa-miR-1282-002803	1.35
hsa-miR-429-001024	3.66	hsa-miR-429-001024	3.25	hsa-miR-15a-000389	-2.18	hsa-miR-142-5p-002248	-2.40
hsa-miR-452-002329	3.00	hsa-miR-452-002329	2.79	hsa-miR-188-3p-002106	-15.67	hsa-miR-144#-002148	-2.95
hsa-miR-484-001821	2.01	hsa-miR-484-001821	2.02	hsa-miR-195-000494	-2.89	hsa-miR-152-000475	2.3
hsa-miR-505#-002087	-2.84	hsa-miR-505#-002087	-1.68	hsa-miR-19a-000395	-2.14	hsa-miR-183#-002270	1.77
hsa-miR-511-001111	3.25	hsa-miR-511-001111	2.16	hsa-miR-21-000397	1.49	hsa-miR-184-000485	-5.46
hsa-miR-708-002341	2.26	hsa-miR-708-002341	2.14	hsa-miR-219-2-3p-002390	26.64	hsa-miR-18a#-002423	1.84
hsa-miR-886-3p-002194	3.00	hsa-miR-886-3p-002194	3.04	hsa-miR-342-5p-002147	-3.96	hsa-miR-200a-000502	3.32
hsa-miR-944-002189	6.72	hsa-miR-944-002189	7.17	hsa-miR-374-000563	-2.24	hsa-miR-223#-002098	-2.63
hsa-miR-1180-002847	3.95	hsa-let-7g-002282	-1.85	hsa-miR-423-5p-002340	-5.05	hsa-miR-299-5p-000600	4.75
hsa-miR-1227-002769	4.37	hsa-let-7i#-002172	-2.59	hsa-miR-486-3p-002093	-4.97	hsa-miR-320-002277	1.92
hsa-miR-130b-000456	1.86	hsa-miR-1244-002791	2.49	hsa-miR-490-001037	3.37	hsa-miR-429-001024	2.37
hsa-miR-135b#-002159	5.18	hsa-miR-138-002284	-3.48	hsa-miR-516-3p-001149	-12.45	hsa-miR-484-001821	2.40
hsa-miR-136#-002100	-3.36	hsa-miR-141#-002145	2.64	hsa-miR-566-001533	-4.10	hsa-miR-532-001518	-2.24
hsa-miR-139-3p-002313	-2.80	hsa-miR-146b-3p-002361	1.29	hsa-miR-583-001623	12.93	hsa-miR-550-002410	-2.05
hsa-miR-142-3p-000464	-3.85	hsa-miR-151-3p-002254	1.69	hsa-miR-640-001584	-4.53	hsa-miR-629-001562	-2.19
hsa-miR-182-002334	3.36	hsa-miR-151-5P-002642	-1.96	hsa-miR-660-001515	-1.96	hsa-miR-663B-002857	1.78
hsa-miR-1825-002907	8.50	hsa-miR-15b#-002173	4.90	hsa-miR-663B-002857	2.55	hsa-miR-888-002212	-551.31
hsa-miR-191#-002678	4.11	hsa-miR-15b-000390	1.89	hsa-miR-942-002187	-3.76		
hsa-miR-197-000497	1.72	hsa-miR-199a-3p-002304	-1.59				
hsa-miR-204-000508	-5.28	hsa-miR-21-000397	2.68				
hsa-miR-211-000514	34.58	hsa-miR-214#-002293	-1.74				
hsa-miR-221-000524	2.69	hsa-miR-222-002276	2.04				
hsa-miR-27b#-002174	1.88	hsa-miR-302c-000533	18.15				
hsa-miR-302a-000529	222.12	hsa-miR-30b-000602	-1.76				
hsa-miR-30a-3p-000416	-3.27	hsa-miR-32-002109	-2.47				
hsa-miR-30a-5p-000417	-1.93	hsa-miR-342-5p-002147	-7.89				
hsa-miR-30e-3p-000422	-2.39	hsa-miR-34b-002102	2.35				
hsa-miR-335-000546	2.33	hsa-miR-520g-001121	3.52				
hsa-miR-34a#-002316	1.91	hsa-miR-577-002675	-3.38				
hsa-miR-378-000567	-1.87	hsa-miR-596-001550	-2.00				
hsa-miR-431-001979	16.66	hsa-miR-649-001602	3.05				
hsa-miR-486-001278	-3.26	hsa-miR-9-000583	5.27				
hsa-miR-486-3p-002093	-3.79						
hsa-miR-497-001043	-4.12						
hsa-miR-517a-002402	3.23						
hsa-miR-517c-001153	3.66						
hsa-miR-523-002386	10.93						
hsa-miR-576-3p-002351	8.52						
hsa-miR-627-001560	29.22						
hsa-miR-643-001594	2.71						
hsa-miR-658-001513	2.12						
hsa-miR-659-001514	6.10						
hsa-miR-99b#-002196	-4.26						

We performed additional analysis and identified 16 miRNAs commonly detected in both type of material and using both type of normalization (Table 4). These miRNAs were screened in database miRSearch V3.0 (Exiqon). Most of them have experimentally proved target genes participating in signaling pathways and cellular processes including not only tumor development. Noteworthy, target genes are transcription factors E2F, MAP kinases, members of RAS protein family, tumor suppressor PTEN, transforming growth factors TGFs, tumor suppressor TP53, proteins participating in cell cycle (cyclins, cyclin-dependent kinases) or regulating cell death.

**Table 4:** Differentially expressed miRNA in both types of clinical material by using two different normalization methods.

hsa-miR-126#-000451	hsa-miR-196b-002215	hsa-miR-21#-002438	hsa-miR-27a-000408
hsa-miR-140-3p-002234	hsa-miR-200b-002251	hsa-miR-210-000512	hsa-miR-29a-002112
hsa-miR-141-000463	hsa-miR-200c-002300	hsa-miR-224-002099	hsa-miR-452-002329
hsa-miR-143-002249	hsa-miR-205-000509	hsa-miR-27a#-002445	hsa-miR-886-3p-002194

**Fig 5:**
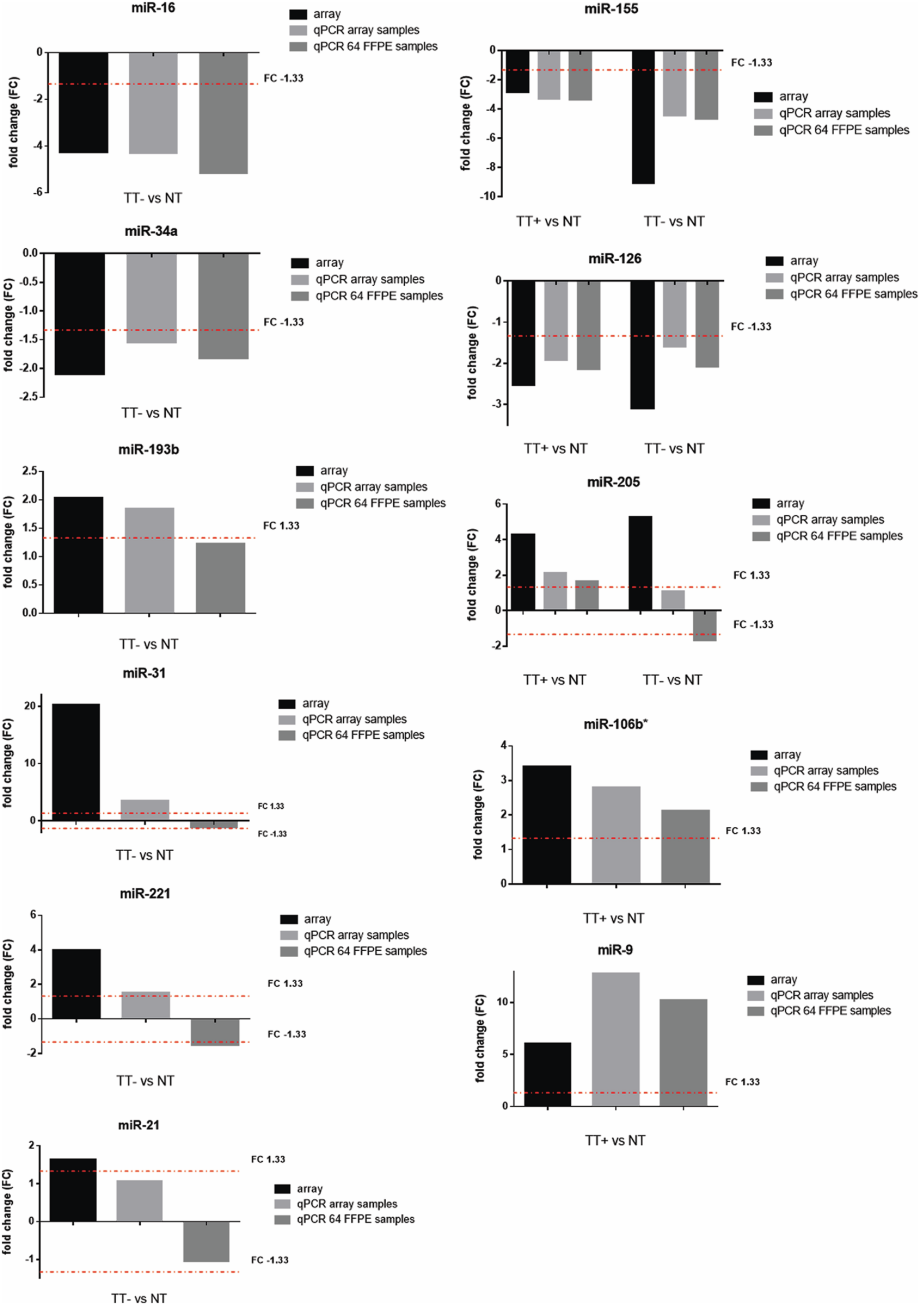
Comparison on fold-change for selected miRNAs between arrays and qPCR. The treshold of FC was set to 1.33. T-test or Mann Whitney nonparametric test was applied depending on the data distribution. All results were statistically significant (P-value ≤0.05).

Finally, from the deregulated miRNAs in FFPE samples, we have selected 11 tumor-specific miRNAs, also with regard to viral or non-viral etiology of the tumor. The expression of these miRNAs was evaluated in a larger set of 64 macrodissected tumor samples by individual TaqMan assays (Fig 5). MiR-106b# and miR-9 were selected as specific for HPV-positive tonsillar tumors, miR-16, miR-34a, miR-193b, miR-31, miR-221, and miR-21 as specific for HPV-negative tumors, and miR-155, miR-126, and miR-205 as specific for tonsillar tumors of any etiology. The differential expression of six miRNAs (miR-106b#, miR-9, miR-16, miR-34a, miR-155, and miR-126) (*P*<0.05; FC>1.33) was confirmed in a larger set of 64 tumor samples, the fold change of miR-193b was equal to 1.23, but the trend of expression was also maintained. The trend of expression of four miRNAs (miR-31, miR-221, miR-21, and miR-205) analyzed in the large set of samples was opposite to that derived from the results revealed by arrays.

## Discussion

The archives of formalin-fixed paraffin-embedded material in pathology laboratories offer the possibility to analyze the samples retrospectively and provide the option for clinical cancer research to extend the studies, and, moreover, to correlate the results with clinical data obtained during long-term follow-up of patients. The utilization of FFPE material for RNA based studies is still challenging, especially due to RNA fragmentation. However, the small size of miRNAs contributes to their stability during the processing of FFPE samples [9] so that FFPE tissues are applicable for miRNA expression analysis studies as previously shown [16–19]. Chatterjee et al. have presented a cross comparison of miRNA detection technologies in FFPE samples and revealed that it is possible to obtain high quality sequence reads for miRNA profiling in FFPE samples with an RNA integrity number around 2 [20].

In our study, we performed the miRNA expression profiling by TLDA analysis in a set of macrodissected FFPE samples and compared the results with the miRNA expression profiles of paired fresh frozen samples. Most studies comparing the miRNA expression between FF and FFPE samples were performed using only several selected miRNAs [21–25].

A comparison of the miRNA expression profiles in FF and FFPE samples obtained by the microarray analysis has also been published. Romero-Cordoba et al. have performed a comparative analysis of paired samples of breast cancer using the TaqMan Low Density Array platform, similarly to Hui et al. [12, 14]. They revealed a high correlation; however, both studies analyzed small groups of paired samples and did not use dissected samples. Goswami et al. have also observed a high correlation between the samples; however, their workflow differed in several aspects (assessment of extraction method, platform optimization, etc.) [13]. Additional studies were performed using other microarray platforms [9, 26, 27] or next generation sequencing platforms [28]. None of this comparative studies has been performed in head and neck tumors.

Since our recent study has shown that the tumor tissue homogeneity is important for the robustness of the miRNA profiling studies, we performed macrodissection of FFPE samples to enrich them for tumor cells. To our knowledge, only one study dealt with the issue of FFPE dissection. It tested the possibility of a non-satisfactory correlation of the expression of several miRNAs between FF and dissected FFPE samples due to the enrichment of the tumor cells. However, the improvement in the correlation when using non-dissected FFPE samples was due to a low number of samples which was not convincing [25]. In our analysis, we compared FF tumor samples with more than 50% of tumor cells and macrodissected FFPE tumor cells. Although, based on the PCA, the miRNA expression profiles differ between FF and FFPE, we performed the Spearman correlation test which showed a good correlation (Spearman correlation coefficient in the range from 0.65 to 0.84) in 70% of samples. Based on the study of de Biase et al. [25] who have pointed to the fact that dissection can influence the expression results which is necessary to take into account if the miRNA analysis is performed with or without dissection, we evaluated the correlation of the differentially expressed miRNAs in FF and FFPE based on the number of tumor cells in FF samples. However, no relationship between the number of tumor cells and correlation level was observed.

We revealed higher numbers of differentially regulated miRNAs in FF tonsillar tissues compared to the FFPE material which we attributed to the higher amount of RNA used for the miRNA expression analysis in FF samples. Therefore, some less expressed miRNAs could have been missed in FFPE samples. The overlap of differentially expressed miRNAs between FF and FFPE samples was around 30% which should be explained by the fact that unlike the tumor samples, the control non-malignant tissues were not paired between FF and FFPE and could influence the miRNA expression profiles more than we expected. Nevertheless, the analysis of both groups of tested material revealed the deregulation of the known tumor associated miRNAs such as miR-205, miR-210, or miRNAs from the family mir-8, as has been demonstrated in a number of solid tumors [29–32].

Further, when comparing the results obtained using two different types of data normalization, we revealed an overlap of between 58–67%. Therefore, the comparison between studies is obviously difficult because of the use of different methods of data analysis. In our analysis, both tissue types and both normalization methods overlap in 16 common miRNAs. All of these miRNAs were shown to be cancer related, nevertheless their role in head and neck cancer has not been elucidated. The role of miR-27a was mentioned by Venkatesh et al. as it regulates the expression of tumor suppressors in oral squamous cell carcinomas [33]. The role of miR-126# was found in endothelial proliferation [34, 35]. For miR-140-3p it was shown that it contributes to reduction of proliferation and migration of cancer cells in breast and lung cancer [36, 37] and in spinal chordoma, in contradictory, to be a marker of poor prognosis [38]. MiR-141 plays role in AKT signaling and inhibits prometastatic mesenchymal characteristics [39]. MiR-143 is a part of miR cluster 143/145 expressed in many tissues and regarded as tumor suppressing [40]. It´s reduced expression was found also in cervical squamous cell carcinomas [41] and low expression of this miRNA contributes to poor prognosis in oral squamous cell carcinomas [42]. As recently discovered the expression of miR-196b promotes cell migration and invasion in oral cancer [43, 44]. Members of mir-200 family, which includes miR-200b, miR-200c and miR-205, are the major regulators of EMT pathway, primarily targeting transcriptional factors ZEB1 and SP1 [45], and play a prognostic role in various malignancies [46]. Deregulated expression of hypoxia-induced miR-210 was found in various tumors and influences cancer cell proliferation, apoptosis, angiogenesis and other cellular processes involved in tumor development [47]. The group of Seki et al. have been interested in the functional significance of mir-29 family, including miR-29a, in cervical cancer as well as in head and neck cancer. They found out that this tumor-suppressive miRNA inhibits cancer cell migration and invasion [48, 49]. Although some studies focused on the functional impact of identified miRNAs in HNC have been published, more extensive research is necessary to more accurately explain the relevance in HNC tumor development.

From the groups of deregulated miRNAs identified in FFPE tumors of either viral or non-viral etiology, we selected 11 miRNAs based on the results and relevance in the literature, the expression of which was evaluated in a larger set of 64 FFPE samples. From the group of miRNAs specific for HPV-positive tonsillar tumors, we choose upregulated miR-9 and miR-106b# whose deregulation was previously reported in HNC [50–52]. Furthermore, miR-9 has been shown to be activated by HPV leading to increased cell motility [53] and to be involved in the pathways regulating metastasis [54]. Upregulated miR-31, miR-221, and miR-21 were selected for confirmation from the group of miRNAs specific for HPV-independent tumors including HNC as playing a role in increasing cell proliferation, invasion, and migration [55–58], participating in regulation of epithelial-mesenchymal transition (EMT), or having a prognostic impact [59, 60]. Further choices were miR-34a, specific for HPV-negative tumors, a tumor suppressor whose downregulation has been shown in a number of tumor types including HNC [61, 62], as well as miR-16, the inhibition of which promotes cell proliferation, migration, invasion, and EMT and contributes to tumor progression [63]. The last miRNA selected for confirmation from the group of HPV-negative tumors was miR-193b whose high expression in tissue was identified as an independent prognostic risk factor in patients with ovarian cancer [64]. From the group of tonsillar specific miRNAs regardless of the virus presence, we selected miR-205, miR-155, and miR-126 which participate in oncogenic pathways including cell proliferation, migration, or invasion and serve as prognostic predictors of patients with HNC [31, 65, 66].

We were able to confirm deregulated expression in the same set of samples as run on array. In this subset, deregulated expression was confirmed for all miRNAs except miR-21 and miR-205. However, their trend of expression was maintained and the fold change was close to the cut-off value. In the larger set of samples, deregulated expression was confirmed in 64% of comparisons. The fold change of miR-193b was equal to 1.23, and the trend of expression of four additional selected miRNAs was opposite to that revealed by arrays. The results suggest possible variability between samples and show that not all deregulated miRNAs detected in a smaller tested cohort might be representative of a broader spectrum of samples.

In conclusion, our study compares the miRNA expression profiles in tonsillar tumors as identified in fresh frozen and formalin-fixed paraffin-embedded samples. Although the correlation between the defined groups was relatively good for all miRNAs, the overlap of the selected differentially expressed miRNAs was rather suboptimal. We concluded that for accurate comparison between studies, the key factors are the use of the same type of clinical material and selection of the normalization method for data analysis. Additionally, we suggest that combining multiple analytical methods rather than a single test alone is advisable for more robust, reliable detection of differential abundance of miRNAs and interpretation of results.
